# Editorial: Focus on spotted lanternfly

**DOI:** 10.3389/finsc.2023.1292590

**Published:** 2023-10-03

**Authors:** Houping Liu, Xiaoyi Wang, Miriam F. Cooperband

**Affiliations:** ^1^ Pennsylvania Department of Conservation and Natural Resources, Harrisburg, PA, United States; ^2^ Ecology and Nature Conservation Institute, Chinese Academy of Forestry, Beijing, China; ^3^ Forest Pest Methods Laboratory, USDA-APHIS-PPQ S&T, Buzzards Bay, MA, United States

**Keywords:** *Lycorma delicatula*, biology, damage, survey and detection, pathogen

The spotted lanternfly (SLF), *Lycorma delicatula* (White) (Hemiptera: Fulgoridae), is a univoltine generalist pest of tree of heaven (TOH) (*Ailanthus altissima* (Mill.) Swingle [Sapindales: Simaroubaceae]) from China (1). As an invasive species, it was found in South Korea in 2004 (2, 3) and most recently Berks County, Pennsylvania in 2014 (4). The current distribution of this pest in the United States includes 14 states from Massachusetts to Indiana and New York to North Carolina (5). In addition to TOH, it also feeds on grapevines and >100 other plant and tree species (6). Large amounts of honeydew excreted during the feeding process promote sooty mold on trunk and leaf surfaces of host trees and understory plants, hindering photosynthesis and contaminating agricultural and forest crops (7). Significant damage in vineyards has been recorded in South Korea (8) and the United States (9). It is a serious threat to the collective multibillion-dollar grape, fruit, nursery, landscape, and hardwood industries in North America and around the world (10, 11).

Managing SLF populations in the field is difficult since TOH is one of the most widespread invasive alien plant species in the United States (12), where more than 400,000 hectares of grapes are cultivated each year (13). Despite concerted efforts on the study of its genetics, host range, behavior, seasonal dynamics, life cycle, dispersal potential, natural enemies, and chemical control by researchers in recent years (14), questions on effective rearing methodology, host-plant interaction, damage, detection, population ecology, behavior, communication, mortality factors, and biological control still remain. The lack of rapid and accurate survey tools creates problems for quality decision-making. Host-switching during the season further complicates population monitoring as different life stages can survive on multiple hosts. Long-distance migration may render localized eradication and control attempts ineffective. Limitations in available management options make infestation containment and population control over large areas almost impossible. However, field populations continue to expand as the result of natural dispersal and human-aided spread. Factors affecting population trends require better understanding. Cutting edge research and paradigm shifting approaches are urgently needed to prevent new infestations from establishing and to mitigate economic loss in currently infested areas.

This Research Topic addresses current knowledge gaps in biology, ecology, and management of SLF through the collection of 20 outstanding articles generated by various research groups in the forefront of the struggle from the United States and China.

To meet the challenge of establishing and maintaining a productive colony for biological control programs and other studies, Nixon et al. successfully developed a rearing method for SLF from newly hatched nymphs to adults based on potted TOH trees in the laboratory. Reliable oviposition was also achieved for females feeding on saturated TOH logs under reduced daylength.

SLF life history traits on different hosts were also studied. Laveaga et al. reported faster development and better survival for nymphs fed on a mixed diet of Concord grape (*Vitis labrusca* L. [Vitales: Vitaceae]) and TOH, as compared with TOH or Concord grape alone, which resulted in significantly heavier adults and more egg masses produced by females. Kreitman et al. observed higher survivorship and shorter development time for nymphs reared on TOH or Concord grape compared with weeping willow (*Salix babylonica* L. [Malpighiales: Salicaceae]), Oriental bittersweet (*Celastrus orbiculata* Thunb. [Celastrales: Celastraceae]), and multiflora rose (*Rosa multiflora* Thunb. [Rosales: Rosaceae]). No nymphs reached adulthood when fed on red maple (*Acer rubrum* L. [Sapindales: Sapindaceae]) whereas those reared on basil (*Ocimum basilicum* L. [Lamiales: Lamiaceae]) failed to complete the second instar. On the other hand, survival and development of both nymphs and adults on specialty crops and other secondary hosts were investigated by Elsensohn et al. Results showed that young (first to second instar) nymphs survived better on Cascade hop (*Humulus lupulus* L. ([Rosales: Cannabaceae]) and adults persisted the best on kiwifruit (*Actinidia* sp. [Ericales: Actinidiaceae]) and muscadine grape (*Vitis rotundifolia* Michx. [Vitales: Vitaceae]). Furthermore, muscadine grape alone could support spotted lanternfly through adulthood, but black walnut (*Juglans nigra* L. [Fagales: Juglandaceae]) needed to pair with TOH for improved survival and development.

Despite the apparent polyphagy for most of its life history, SLF does aggregate as adults towards TOH for defense sequestration, maturation feeding, and mating (15–17). Cooperband and Murman captured this behavior in the field when they found males were generally attracted to males while females to females in their cage studies, resulting in male-biased, sex-balanced, and female-biased adult populations on TOH trees in the early, mid-, and late stages, respectively. Further investigation by Faal et al. through olfactometer trials in the laboratory revealed that honeydew volatiles such as 2-heptanone, 2-octanone, and 2-nonanone were likely responsible for this kind of behavior. Along with plant volatiles discovered before (18), they could potentially be used as lures for the development of adult traps down the road.

SLF egg masses are found on the surfaces of a wide range of substrates from trees to plants and nonliving materials (1, 16, 17). However, oviposition is not completely random but rather selected by habitats and substrates as demonstrated by Liu. Egg mass size ranged from 0-105 eggs/mass with larger egg masses found in newly infested sites with expanding populations. Egg masses laid on American beech (*Fagus grandifolia* Ehrh. [Fagales: Fagaceae]), black birch (*Betula lenta* L. [Fagales: Betulaceae]), black cherry (*Prunus serotina* Ehrh. [Rosales: Rosaceae]), black locust (*Robinia pseudoacacia* L. [Fabales: Fabaceae]), hackberry (*Celtis occidentalis* L. [Rosales: Cannabaceae]), Norway maple (*Acer platanoides* L. [Sapindales: Sapindaceae]), pawpaw (*Asimina triloba* (L.) Dunal [Magnoliales: Annonaceae]), red maple, and sweet cherry (*Prunus avium* L. [Rosales: Rosaceae]) generally hatched better than those on other substrates.

Three studies focused on the impact of SLF feeding on its hosts. Dechaine et al. reported diminished annual diameter growth on infested TOH trees based on dendrochronological evidence after data were standardized by climate variables and tree size and age. However, no such impact was observed on infested black walnut and tuliptree (*Liriodendron tulipifera* L. [Magnoliales: Magnoliaceae]). Chemical treatment of SLF with dinotefuran through basal bark application did not help TOH growth compared with untreated controls. Islam et al. discovered genome-wide transcriptional response on grapes after heavy SLF feeding, with extensive changes in gene expression in pathways associated with biosynthesis of lignin and other structural components of cell-wall matrix and antioxidant/detoxification. Lavely et al. recorded negative impact of short-term feeding by late-stage nymphs and adults on the carbon assimilation for red maple and silver maple (*Acer saccharinum* L. [Sapindales: Sapindaceae]) in a common garden study, with no significant negative impact from SLF feeding on black walnut.

Without effective prevention and management measures, SLF has the potential to establish in most New England, mid-Atlantic, Midwest, and Pacific Coast states in the United States in addition to other suitable areas around the world (19, 20). Keena et al. predicted even wider potential climate range for SLF in North America based on upper (T_max_) and lower (T_min_) developmental thresholds in areas previously considered too cold to be at risk, while southward expansion into warmer regions may be limited by thermal conditions as forecasted by those models.

Accurate population estimation is difficult for SLF because of the high mobility of nymphs and adults and diverse habitats of egg masses. Lewis et al. developed an effective and low-cost lamp shade trap for egg masses after tens of designs and hundreds of field deployments in five years. This design could revolutionize SLF population monitoring and egg mass collection in the field with an average of 25 (up to 111) egg masses recovered from each trap. Guidelines for trap construction and its field installation were also included as supplementary materials for this publication. On the other hand, Belouard and Behm introduced a new way to identify adults by wing spot patterns using computer-aided photo-identification technology. After validation by larger datasets, it could have broad applications in population survey and dispersal studies for SLF adults without laborious work on marking, releasing, and recapturing of the insects.

Many chemical insecticides (e.g., chlorpyrifos, thiamethoxam, bifenthrin) are effective against SLF eggs, nymphs, and adults through topical application (21, 22). In places when cover spray is not desired, systemic insecticides can be used as the alternatives. Keyzer et al. demonstrated that dinotefuran could persist on TOH bark surface for at least 100 days when applied through basal trunk spray, providing effective management for SLF adult populations in the field.

Major mortality factors for SLF in the field include predators, parasitoids, and pathogens (23–28). Multiple strains of *Beauveria bassiana* Bals. -Criv) Vuill. (Hypocreales: Cordyciptaceae) were isolated by Clifton et al. from field infected SLF in Pennsylvania, with two of the most prevalent strains showing superior pathogenicity against nymphs and adults than a standard commercial strain in laboratory bioassays.

*Anastatus orientalis* Yang & Choi (Hymenoptera: Eupelmidae) is a solitary egg parasitoid of spotted lanternfly in its native range (25–27). Its potential as a biological control agent for SLF in North America is being considered. By analyzing mitochondrial DNA, Wu et al. were able to recover six haplotypes of *A. orientalis* based on specimens collected from China and South Korea, with haplotypes B, C, and D widely distributed while haplotypes A, E, and F found only in certain locations. Bao et al. investigated the impact of photoperiod experience and found increased fecundity and female-based sex ratio, but decreased longevity in the next two generations when parents were placed under long daylength. The broad host range of haplotype C was confirmed after choice and no-choice testing against 36 eastern species in 18 families by Broadley et al. and 34 southwestern species in 12 families by Gómez-Marco et al, with nontarget species in the families of Coreidae, Erebidae, Fulgoridae, Lasiocampdae, Pentatomidae, Saturnidae, and Sphingidae readily attacked. For laboratory rearing purpose, Gómez-Marco and Hoddle found that exposing SLF eggs to -40 °C for more than 1 h effectively killed SLF eggs, however, *A. orientalis* females could still utilize them to produce progenies successfully. The potential of this egg parasitoid as an SLF biocontrol agent would rest on other haplotypes if they are found to have narrower host ranges.

Successful management of SLF in North America depends on the integration of new information from sound scientific research for early detection and rapid response activities. New findings in laboratory rearing, life history characteristics, adult behavior, host damage, potential climate range, novel survey and detection tools, pathogens and parasitoids, and chemical control in this Research Topic could help decision makers to refine their management strategies. At the same time, research community could also benefit from each study in its pursuit of better outcomes in various areas. The potential damage by SLF to other specialty crops and hardwood trees is not expected to approach the level on grapes. SLF management on grapes should focus on chemical control of SLF adults feeding on vines after emergence and those aggregating on TOH trees in the surrounding areas before mating, with microbial control of high density nymphal and adult populations and biological control being parts of the solution. Conversely, homeowners and residents in urban and suburban areas could also take advantage of the new information provided by the studies to mitigate the impact of this significant nuisance and improve their quality of life.

## Author contributions

HL: Writing – original draft, Writing – review & editing. XW: Writing – review & editing. MFC: Writing – review & editing.
